# Epidemiology of co-infections in pregnant women living with human immunodeficiency virus 1 in rural Gabon: a cross-sectional study

**DOI:** 10.1186/s40249-023-01114-y

**Published:** 2023-07-06

**Authors:** Saskia Dede Davi, Dearie Glory Okwu, Marc Luetgehetmann, Frederique Mbang Abba, Martin Aepfelbacher, Lillian Rene Endamne, Ayodele Alabi, Rella Zoleko-Manego, Ghyslain Mombo-Ngoma, Saidou Mahmoudou, Marylyn Martina Addo, Michael Ramharter, Johannes Mischlinger

**Affiliations:** 1 Centre of Tropical Medicine, Bernhard-Nocht Institute for Tropical Medicine and I. Department of Medicine, University Medical Center Hamburg-Eppendorf, Hamburg, Germany; 2 German Centre for Infection Research, Partner Site Hamburg-Lübeck-Borstel-Riems, Hamburg-Lübeck-Borstel-Riems, Hamburg, Germany; 3 Centre de Recherches Médicales de Lambaréné, Lambaréné, Gabon; 4 Center for Diagnostics, Institute of Medical Microbiology, Virology and Hygiene, University Medical Center Hamburg-Eppendorf (UKE), Hamburg, Germany; 5 Albert-Schweitzer Hospital, Lambaréné, Gabon; 6 Department of Parasitology, Leiden University Medical Center, Albinusdreef, The Netherlands; 7 Department for Clinical Immunology of Infectious Diseases and I. Department of Medicine, University Medical Center Hamburg-Eppendorf, Hamburg, Germany

**Keywords:** HIV, Cross-sectional study, Pregnancy, Sexually transmitted infections, Gabon

## Abstract

**Background:**

There is no recent epidemiological data on HIV infection in Gabon, particularly in pregnant women. To close this gap, an HIV-prevalence survey was conducted among Gabonese pregnant women, followed by a cross-sectional case–control study in which the prevalence of various co-infections was compared between HIV-positive and HIV-negative pregnant women.

**Methods:**

Between 2018 and 2019, data for the HIV-prevalence survey were collected retrospectively in 21 Gabonese antenatal care centres (ANCs). Subsequently, for the prospective co-infection study, all HIV-positive pregnant women were recruited who frequented the ANC in Lambaréné and a comparator sub-sample of HIV-negative pregnant women was recruited; these activities were performed from February 2019 to February 2020. The mean number of co-infections was ascertained and compared between HIV-positive and HIV-negative women. Additionally, the odds for being co-infected with at least one co-infection was evaluated and compared between HIV-positive and HIV-negative women.

**Results:**

HIV-positivity was 3.9% (646/16,417) among pregnant women. 183 pregnant women were recruited in the co-infection study. 63% of HIV-positive and 75% of HIV-negative pregnant women had at least one co-infection. There was a trend indicating that HIV-negative women were more often co-infected with sexually transmitted infections (STIs) than HIV-positive women [mean (standard deviation, *SD*): 2.59 (1.04) vs 2.16 (1.35), respectively; *P* = 0.056]; this was not the case for vector-borne infections [mean (*SD*): 0.47 (0.72) vs 0.43 (0.63), respectively; *P* = 0.59].

**Conclusions:**

Counterintuitively, the crude odds for concomitant STIs was lower in HIV-positive than in HIV-negative women. The change of magnitude from the crude to adjusted *OR* is indicative for a differential sexual risk factor profile among HIV-positive and HIV-negative women in this population. This might potentially be explained by the availability of sexual health care counselling for HIV-positive women within the framework of the national HIV control programme, while no such similar overall service exists for HIV-negative women. This highlights the importance of easy access to sexual healthcare education programmes for all pregnant women irrespective of HIV status.

## Background

Approximately 38.4 million individuals live with the Human Immunodeficiency Virus (HIV) worldwide. The burden of HIV infection is highest in sub-Saharan Africa (SSA), where 66.7% of all globally infected individuals live. According to UNAIDS, in 2021, more than half (54%) of all individuals living with HIV worldwide were women and girls [1, 2].

Infection with HIV produces cellular immune deficiency and increases the susceptibility to various co-infections [3]. Co-infections in HIV-infected pregnant women may increase the risk of mother-to-child-transmission (MTCT) of HIV [4], and co-infections targeting the placenta and the birth tract are more likely to interfere with the infant’s organogenesis, where they can cause adverse birth events [5].

Gabon has a national HIV/AIDS control programme offering screening for HIV among antenatal care (ANC) unit attendees. The programme collects data on HIV prevalence in pregnant women to strengthen the prevention of MTCT of HIV. During the first antenatal care visit, the pregnant women are systematically screened for HIV using rapid diagnostic tests according to the national guidelines. However, few data characterise the most frequent co-infections among HIV-positive women during pregnancy compared to HIV-negative pregnant women in Gabon. To close this gap, we first conducted a retrospective study to determine the prevalence of HIV among pregnant women in Gabon and second a comparative study in which co-infections were assessed and compared between HIV-positive and HIV-negative pregnant women. These data may provide feedback to the national programme for mother and child health to discuss relevant outcomes with concerned stakeholders on a regional and national level and to improve mother and child healthcare in Gabon.

## Methods

### Study design and study population

The project consisted of two studies, first, the HIV prevalence study and second, the co-infection study. For the HIV prevalence study, representative ANC units were identified where pregnant women of any age received routine care. There, HIV prevalence was determined retrospectively using routinely collected diagnostic data. Due to the ongoing activities of the Gabonese National HIV/AIDS Control programme, data on the frequency of first visits, the number of women tested for HIV, and those who tested HIV positive were accessible at each ANC unit. For the co-infection study, a cross-sectional survey was performed assessing the prevalence of co-infections in a sample of HIV-positive and a comparator sub-sample of HIV-negative pregnant women. A pregnant woman was eligible if she gave written consent and if her HIV status was known.

### Study sites

The HIV prevalence study included pregnant women of all ages who attended 21 antenatal care (ANC) centres in Gabon: Fougamou Hospital, Albert Schweitzer Hospital, Regional Hospital of Melen, Nzeng-Ayong Hospital, Health Centre Akebe-Plaine, Health Centre Lalala, Chinese-Gabonese Hospital, Health Centre of London, Health Centre of Glass, Health Centre of Louis, Health Centre of the Peyrie, Health Centre of Awendje, Community Health Centre in Libreville, University Medical Centre of Owendo, Health Centre of Egypto, Mother-and-Child-Health of Tchibanga, Medical Centre of Mayoumba, Regional Medical Centre of Oyem, Regional Unit for Mother-and-Child Health of Oyem, Medical Centre of Bitam. It was conducted between 2018 and 2019. Targeted ANCs were located in five out of nine provinces (Moyen-Ogooué, Nyanga, Ngounié, Estuaire, Woleu Ntem) in Gabon, Central Africa. The co-infection study was conducted between February 2019 and February 2020 in Lambaréné, Gabon. Lambaréné is a semi-urban town in Moyen-Ogooué and was chosen for the co-infection study based on a regionally high prevalence of HIV 5.2% (18/345) and easy geographical accessibility for the study team. The health facilities used for this study were the Albert Schweitzer hospital and the Antenatal Care Unit in Lambaréné.

### Data and sample collection

Socio-demographic and obstetrical data were collected. Obstetrical data included: gestational age, gravidities, and parties. The prevalence of several viral infections was determined: HIV-2, human T-lymphotropic virus type 1 and 2 (HTLV-1 and 2), hepatitis B virus (HBV), hepatitis E virus (HEV), hepatitis C virus (HCV), Epstein-Barr virus (EBV), and human papillomavirus (HPV). Infection with a high-risk subtype of HPV was defined as the presence of at least one of the following subtypes: 16, 18, 31, 33, 34, 35, 39, 45, 51, 52, 56, 58, 59, 66, 68, and 70; all other HPV subtypes were considered as HPV low-risk subtypes [6]. Moreover, the following bacterial infections were determined: *Neisseria gonorrhoea* (NG), *Chlamydia trachomatis* (CT), *Treponema pallidum* (TP), Group B *Streptococcus* (GBS). The following parasitic infections were assessed: *Plasmodium* spp., *Loa loa* microfilariae, *Mansonella perstans* microfilariae, *Trichomonas vaginalis* (TV), *Schistosoma haematobium*. All HIV-positive women were invited to participate, and age and parity-matched HIV-negative women were offered participation as controls. Matching was considered successful if the median age difference between HIV-negative and HIV-positive women was ≤ 4 years and the difference in parity ≤ 1, respectively.

### Assessment of co-infections

As HIV-1 and 2, HBV and TP testing was included in the routine diagnostic services according to the Gabonese National HIV/AIDS Control Programme, and test results were transcribed from clinical records. CD4 count was also often available for HIV-positive women as part of routine diagnostic services in Gabon, and was systematically recorded this information. To determine infection with HPV, TV, CT, NG and GBS endocervical, ectocervical and recto-vaginal samples using nylon swabs (eNat Copan Diagnostics, Brescia, Italy) that contained a one ml liquid Amies transport medium were collected. An EDTA tube and a serum tube with 4.5 ml peripheral blood were collected to assess various co-infections. All blood samples were directly aliquoted into 2.5 ml Eppendorf Tubes. All cervical and blood samples were conserved at − 80 °C for shipment to the microbiology ward at University Medical Center Hospital Hamburg-Eppendorf (UMCHE) and the Bernhard-Nocht-Institute for Tropical Medicine (BNITM), Germany. At BNITM, *Schistosoma haematobium* deoxyribonucleic acid (DNA) was determined using the Qiagen^®^ kit for the Taqman^®^ qPCR. The presence of *Plasmodium* spp. was determined by microscopic examination of thick and thin blood smears using the Lambaréné method at Centre de Recherches Médicales de Lambaréné (CERMEL) [7]. *Mansonella perstans* and *Loa loa* microfilariae were assessed in peripheral blood by light microscopic examination of thick blood smears at CERMEL. GBS colonisation was determined at CERMEL as described elsewhere [8]. At the microbiology ward of UMCHE, HPV, TV, CT, NG, HTLV 1 and 2, HEV, HCV, and EBV were determined at the department of microbiology at UMCHE using the LightCycler 480II (Roche^®^, Basel, Switzerland) for qPCR. For HPV, an Anyplex II HPV28 (Seegene^®^, Duesseldorf, Germany) determining 28 subtypes was used. Chemiluminescent Immunoassay (CLIA) was done to detect anti-HTLV-1/HTLV-2 antibodies in serum with the system Alinity I (Abott^®^, Abott Park, USA). ELISA was done with the Euroimmun analyser I (Euroimmun^®^, Luebeck, Germany) to determine anti-HEV-IgG and anti-HEV IgM assay in serum using Wantai^®^ (Pecking, China). Anti-HCV antibodies in serum were determined using Alinity I (Abott^®^, Abott Park, USA). In the presence of IgG and IgM antibodies, the sample was considered positive; the absence of IgG and IgM antibodies was considered hepatitis virus negative. HIV-viral load and EBV infection were determined by PCR [9]. Unless otherwise specified, all samples were analysed according to the manufacturer’s instructions*.*

### Data management and statistics

Data was collected using paper-based source documents and transcribed to an electronic database (Redcap, Vanderbilt University, USA). Statistical analyses were conducted using STATA 17.0 Basic Edition (StataCorp, Lakeway Drive, USA). Prevalence was calculated as the number of positive cases divided by the number of pregnant women tested during the study period. For proportions, P-values were determined using the Chi-square ($\upchi$^2^) test. If a pregnant woman had at least one concomitant infection, she was termed positive for “any concomitant infection”. Means and standard deviations (*SD*) were presented for parametrically distributed continuous data and medians, interquartile ranges (IQR) and ranges were used to present non-parametrically distributed continuous data; t-tests and Wilcoxon rank sum tests were used for the hypothesis testing of continuous data with parametric and non-parametric spread, respectively. Logistic regression models were used to compute unadjusted and adjusted odds ratios (*OR*s) and 95% confidence intervals (*CI*s) of any concomitant infection, vector-borne infection, and STI. Age and employment were adjusted for in the ‘any concomitant infection’ and ‘any vector-borne infection’ models. Concerning the ‘any STI’ model, the following variables were adjusted: age, employment, gravidity, sexual partners in life, sexual partners within the previous month, living with a partner, and method of contraception.

## Results

### HIV Prevalence among pregnant women

Overall, 16,417 pregnant women were screened for HIV in 21 ANC units from 2017 to 2018, with the average prevalence being 3.9% (646/16,417). The overall prevalence in the capital, Libreville, was 3.6% (450/ 12,471), ranging between 1.7% (17/1017) and 6% (92/1546). The HIV prevalence in the Northern province, Woleu-Ntem (Oyem and Bitam), was 4.2% (53/1273); in the centrally located Moyen-Ogooué (Lambaréné), 5.2% (18/345); and the Southern provinces of Ngounié and Nyanga, 3.8% (10/263) and 5.6% (33/594), respectively.

### Prevalence of co-infections among pregnant women

One-hundred-and-eighty-three participants were recruited into the co-infection study: 62 HIV-positive and 121 HIV-negative pregnant women. Overall, the median age was 27 (IQR: 22–33) years; HIV-positive pregnant women had a median age of 29 (IQR: 24–35) years and HIV-negative pregnant women had a median age of 25 (IQR: 20–33) years. Median parity was 2 (IQR: 1–3) (Table 1). Parity was higher among HIV-positive pregnant women, with a parity of 3 (IQR: 1–4) compared with a parity of 2 (IQR: 1–3) amongst HIV-negative pregnant women. 19.1% (35/183) were nulliparous in the overall sample.

**Table 1:** Demographic and obstetric characteristics of HIV-positive and HIV-negative pregnant women

Characteristics	Totals (*N* = 183)	HIV+ (*n* = 62)	HIV− (*n* = 121)
	*n* (column %)	*n* (column %)	*n* (column %)
Age			
Median age in years (IQR)	27 (22–33)	29 (24–35)	25 (20–33)
10–19 years	22 (12%)	2 (3.2%)	20 (16.5%)
20–29 years	91 (49.7%)	30 (48.4%)	61 (50.4%)
30 and above	70 (38.3%)	30 (48.4%)	40 (33.1%)
Parity			
Median number of parities (IQR)	2 (1–3)	3 (1–4)	2 (1–3)
Nulliparous	35 (19.1%)	8 (12.9%)	27 (22.3%)
Primiparous	35 (19.1%)	8 (12.9%)	27 (22.3%)
Multiparous (2–5)	92 (50.3%)	38 (61.3%)	54 (44.6%)
Multiparous (> 5)	21 (11.5%)	8 (12.9%)	13 (10.7%)
Any concomitant infection (*n* = 183)			
Negative	53 (29%)	23 (37.1%)	30 (24.8%)
Positive	130 (71%)	39 (62.9%)	91 (75.2%)
Any vector-borne infection (*n* = 175)			87 (71.9%)
Negative	111 (63.4%)	35 (63.6%)	76 (63.3%)
Positive	64 (36.6%)	20 (36.4%)	44 (36.7%)
Any sexually transmitted infection (*n* = 178)			
Negative	81 (45.5%)	34 (59.7%)	47 (38.8%)
Positive	97 (54.5%)	23 (40.4%)	74 (61.2%)

*IQR* interquartile range

The overall proportion of pregnant women with at least one co-infection was 71% (130/183), with a mean number of concomitant infections of 2.92 (*SD* = 1.45). The proportion of HIV-positive pregnant women with at least one infection was 62.9% (39/62), while the proportion of HIV-negative was higher at 75.2% (91/121) (Table 1). The mean number of concomitant infections in HIV-positive women was 2.5 (*SD* = 1.66) and 3.12 (*SD* = 1.29) in the HIV-negative sub-sample (Table 2). Concordantly, the proportion of women without any concomitant infection was larger in the HIV-positive sample than in the HIV-negative sub-sample (37.1% vs 24.8%, respectively).

**Table 2:** Proportion of concomitant infections in HIV-negative and HIV-positive pregnant women

Infection	Totals (*N* = 183)	HIV+ (*n* = 62)	HIV− (*n* = 121)	*P*-value	Name of test
	*n* (column %)	*n* (column %)	*n* (column %)		
Number of any concomitant infection (n = 183)					
Median (IQR)	3 (2–4)	3 (2–4)	3 (2–4)	0.0183	Wilcoxon rank sum test
Mean (*SD*)	2.92 (1.45)	2.5 (1.66)	3.12 (1.29)	0.0095	*t*-test
Negative for all infections	53 (29%)	23 (37.1%)	30 (24.8%)	0.082	Chi^2^ test
Positive for at least one infection	130 (71%)	39 (62.9%)	91 (75.2%)		
Number of vector-borne infections (*n* = 175)					
Median (IQR)	0 (0–1)	0 (0–1)	0 (0–1)	0.87	Wilcoxon rank sum test
Mean (*SD*)	0.43 (0.63)	0.47 (0.72)	0.42 (0.59)	0.59	*t*-test
Negative for all infections	111 (63.4%)	35 (63.6%)	76 (63.3%)	0.97	Chi^2^ test
Positive for at least one infection	64 (36.6%)	20 (36.4%)	44 (36.7%)		
Number of sexually transmitted infections (*n* = 178)					
Median (IQR)	2 (2–3)	2 (2–3)	3 (2–3)	0.056	Wilcoxon rank sum test
Mean (*SD*)	2.45 (1.16)	2.16 (1.35)	2.59 (1.04)	0.021	*t*-test
Negative for all infections	81 (45.5%)	34 (59.7%)	47 (38.8%)	0.009	Chi^2^ test
Positive for at least one infection	97 (54.5%)	23 (40.4%)	74 (61.2%)		

*IQR* interquartile range, *SD* standard deviation

### Description of HIV-positive sample

With regards to the HIV-positive sample, median values for time since HIV diagnosis, CD4 count and viral load were 0.23 (IQR: 0.02–3.5) years, 534 cells/mm^3^ (IQR: 255–688), and 384 (range: 0–654,000) copies/ml (Table 3), respectively. Among HIV-positive participants for whom medication history was available, the majority (60.7%, 37/61) had initiated antiretroviral therapy (ART). Data on co-trimoxazole preventive therapy (CTX) was available for 48 women. 35 out of 48 women (68.8%) for whom data on CTX was available did not take CTX.

**Table 3:** Clinical and biological HIV parameters of HIV-positive pregnant women

Variables	HIV+ (*n* = 62)
	*n* (column %)
Time since HIV diagnosis (*n* = 35)	
Median time in years (IQR)	0.23 (0.02–3.5)
< 1 year	22 (62.9%)
1–4 years	7 (20%)
5–9 years	5 (14.3%)
10 years and more	1 (2.9%)
ART initiated (n = 61)	
Yes	37 (60.7%)
No	24 (39.3%)
Time since ART initiation (*n* = 35)	
Median time in years (IQR)	0.23 (0.02–3.5)
< 1 year	22 (62.9%)
1–4 years	7 (20%)
5–9 years	5 (14.3%)
10 years and more	1 (2.9%)
CD4 counts (*n* = 31) in cells/mm^3^	
Median (IQR)	534 (255–688)
< 50	1 (3.2%)
50–99	1 (3.2%)
100–199	4 (12.9%)
200–299	2 (6.5%)
300–399	4 (12.9%)
400–499	3 (9.7%)
500 and above	16 (51.6%)
CTX taken (*n* = 48)	
Yes	15 (31.3%)
No	33 (68.8%)
Viral load in copies/ml (*n* = 37)	
Median (range)	384 (0–654,000)
0	13 (35.1%)
1–99	2 (5.4%)
100–999	8 (21.6%)
1000–9999	7 (18.9%)
10,000–99,999	4 (10.8%)
> 100,000	3 (8.1%)

*ART* antiretroviral therapy, *CTX* co-trimoxazole preventive therapy, *IQR* interquartile range

### Associations between HIV status and the presence of co-infections

The odds of having any concomitant infection in HIV-positive pregnant women in the unadjusted model was 0.52 times (*OR* = 0.52; 95% *CI*: 0.27–1.00) the odds of HIV-negative women (*P* = 0.052). After adjusting for age and employment status, the adjusted odds of having any concomitant infection in HIV-positive women was 0.64 (95% *CI*: 0.32–1.28), and the association was lost (*P* = 0.21). The most prevalent co-infections were HPV (70.6%, 84/119) and TV (24.4%, 29/119). Among the pregnant women with HIV, 74.1% (20/27) were infected with HPV compared with 69.6% (64/92) among HIV-negative women. Almost three-quarters of HIV-positive pregnant women (72%; 18/25) and approximately two-thirds of HIV-negative women (65%; 52/80) were infected with high-risk HPV subtypes. Having an infection with an HPV high-risk subtype was not associated with HIV status (*P* = 0.5).

**Fig. 1:**
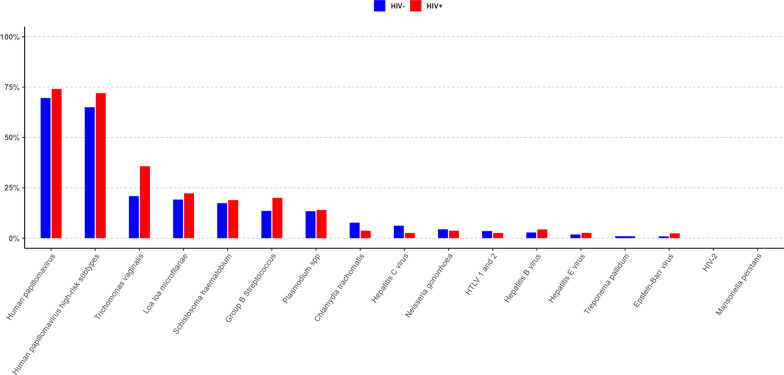
Proportion of various co-infections in HIV-negative (blue) and HIV-positive (red) pregnant women

Among HIV-positive women, 36% (10/28) had an infection with TV, and 21% (64/110) among HIV-negative women were infected with TV (Fig. 1). The prevalence of GBS colonisation in HIV-positive pregnant women was 20% (3/15) and 13.5% (5/37) in HIV-negative pregnant women; it is of mention that GBS samples could be obtained by only a small selection of women due to logistical reasons. Compared to pregnant women with a negative HIV status, pregnant women with a positive HIV status were less likely to have concomitant STIs (*OR* = 0.40; 95% *CI*: 0.21–0.76; *P* = 0.005). When adjusting only for age, the *OR* was 0.42 (95% *CI*: 0.22–0.82, *P* = 0.01). In the adjusted model corrected for age, employment and sexual risk factors, the association of having a concomitant STI became statistically non-significant (*P* = 0.28) (Table 4). There was no evidence of an association between vector-borne infections and HIV status (crude *OR* = 0.99; *P* = 0.97; adjusted *OR* = 1.40; *P* = 0.36; Table 4).

**Table 4:** Associations between concomitant infections and HIV-positivity among pregnant women in Gabon

Infection	HIV+	HIV−	Crude *OR*s (95% *CI*s)	*P* value	Adjusted variables	Adjusted *OR*s (95% *CI*s)	*P* value	Likelihood ratio test (*P* value)
	*n* (column %)	*n* (column %)						
Any concomitant infection								
Negative	23 (37.1%)	30 (24.8%)	1	0.052	Age, employment status	1	0.21	0.21
Positive	39 (62.9%)	91 (75.2%)	0.52 (0.27–1.00)			0.64 (0.32–1.28)		
Any vector-borne infection								
Negative	35 (63.6%)	76 (63.3%)	1	0.97	Age, employment status	1	0.36	0.36
Positive	20 (36.4%)	44 (36.8%)	0.99 (0.51–1.92)			1.40 (0.68–2.88)		
Any sexually transmitted infection								
Negative	34 (59.7%)	47 (38.8%)	1	0.005	Gravidity, number of sexual partners in life, number of sexual partners last month, living with a partner, age, employment status, method of contraception	1	0.28	0.28
Positive	23 (40.4%)	74 (61.2%)	0.4 (0.21–0.76)			0.57 (0.20–1.59)		

*OR* odds ratio, *CI* confidential interval

## Discussion

In this study, we described the HIV prevalence among pregnant women who attended 21 ANC units in 2018 across four provinces in Gabon, Central Africa. The overall prevalence of HIV among pregnant women attending the ANC units in Libreville, Lambaréné, Fougamou, Tchibanga, Mayoumba, Oyem, and Bitam was 3.9% (646/16,417). A comparison of setting-specific HIV prevalence showed moderate disparities between regions and sub-regions in Gabon. However, based on the design of our study, the cause of these regional disparities could not be determined. Yet, these findings should raise awareness among public health officials, community stakeholders, and the Gabonese Ministry of Health concerning a potential inequality of access to the HIV prevention programme during ANC in Gabon. Therefore, to further assess the reasons for this spatial variability of HIV prevalence, further research should be conducted.

Most HIV-positive pregnant women were aware of their HIV status and correctly received ART directly after diagnosis. Moreover, CTX was usually not indicated as the indication for CTX administration in Gabon was a CD4 count < 500/mm^3^. However, CD4 count was not available for half of the HIV-positive pregnant women in this study, 50% (31/62), due to missing data. Therefore, based on the scarcely available CD4-count data, it is challenging to conclude how HIV might have influenced the distribution of co-infections among HIV-positive pregnant women.

Concomitant co-infections were overall more common in HIV-negative than in HIV-positive pregnant women. When restricting to only vector-borne infections, there was no difference among HIV-positive and HIV-negative women [0.99 (95% *CI*: 0.51–1.92)]. In contrast, concomitant STIs were more frequent in HIV-negative pregnant (61.2%, 74/121) women than in HIV-positive women (40.4%, 23/57). This points towards the counterintuitive situation that HIV positivity may have been a protective factor for acquiring STIs in our study population.

In Gabon, ART access for HIV-positive people involves regular visits and counselling by health care providers, including education on condom use and sexual abstinence to reduce transmission risk. Thus, unlike women with a negative HIV status, HIV-positive pregnant women could have been appropriately informed by health care providers about the prevention of transmission of HIV and thereby also been less likely to spread or acquire other STIs [10]. Similar findings were found in a cohort study (*N* = 237 women) by Mukanyangezi et al. conducted in Rwanda, assessing 100 HIV-positive and 137 HIV-negative women. They investigated whether the sexual risk behaviour of women differed according to HIV status. The prevalence of STIs was determined at baseline and nine months of follow-up. They found that HIV-positive women had fewer STIs than HIV-negative women at baseline (26.2% vs 66%, respectively; *P* = 0.001). However, they found that the incidence of STIs among HIV-positive and HIV-negative women did not significantly differ at nine months of follow-up. This might have been due to the Hawthorne effect and increased health awareness of individuals due to participation in this cohort study. The authors concluded that the prevalence of STI was high among Rwandan women and that an HIV-positive status changed sexual risk behaviour. However, they also concluded that having any STI (except for HIV) did not change the sexual risk behaviour [11]. In our study, we determined the prevalence of STIs only at one-time point and do not know if the difference at baseline could have disappeared over time. In Gabon, HIV infection is highly stigmatised [10]. A fact that encourages HIV-positive pregnant women to hide their HIV status. The ultimate fear of stigma and resulting ostracism may reduce risky behaviour associated with the spread of HIV. This, again, would simultaneously reduce the chance of spreading and acquiring other STIs.

Moreover, the high prevalence of STIs among pregnant women in SSA has also been described in different settings than ours [11–16]. Akarolo-Anthony et al. conducted a case–control study in 2012 in Abuja, Nigeria, to assess the association between HIV-positivity and the risk of being infected with HPV high-risk subtypes in women presenting for cervical cancer screening. 54% (151/278) HIV-positive, 40% (111/278) HIV-negative women and 6% (16/278) with an unknown HIV status were included in this study. The authors determined HPV high-risk and low-risk subtypes using PCR and nucleic acid hybridisation. In contrast to our study, they found an increased risk of infection with high-risk HPV subtypes when being HIV-positive in their study population.

In a cross-sectional study conducted from 2017 to 2018 in Cape Town, South Africa, Davey et al. demonstrated a higher prevalence of STIs, including infection with NG, CT, TV, and TP in HIV-positive pregnant women (32%, 42/107) compared to HIV-negative (28%, 38/135) pregnant women (*OR* = 1.65, 95% *CI*: 0.96–2.83) [12]. In our study, the prevalence of GBS colonisation was 15.4% (8/52). In a multicentre randomised clinical trial carried out at CERMEL from April 2010 and January 2012, it was reported that the prevalence of GBS colonisation in the study region was 19% at delivery (95% *CI*: 16–23%; 106 out of 549 participants) [17]. The findings of Capan-Melser et al. were consistent with our study and several different studies, which determined the prevalence of GBS colonisation among pregnant women [17–19]. The global prevalence of GBS, including 85 countries and 300,000 pregnant women, was estimated at 18% (95% *CI*: 17–19%). In this study, HIV-positive pregnant women were as often colonised with GBS as HIV-negative women [(20% (3/15) vs 13.5% (5/37); *P* = 0.6]. As GBS colonisation at delivery is an essential risk factor for adverse birth outcomes, we underline the importance of implementing a GBS prevention programme among pregnant women before and during delivery in Gabon as recommended in other countries [18–23].

Compared to pregnant women with a negative HIV status, those with a positive HIV status were less likely to have concomitant STIs (crude *OR* = 0.40; 95% *CI*: 0.21–0.76). Yet, in the adjusted model, the statistical association of having a concomitant STI became statistically non-significant (adjusted *OR* = 0.57; 95% *CI*: 0.20–1.59). This change between the crude and the adjusted *OR* indicates the presence of positive confounding, posed mainly by sexual risk factors. Thus, the above-mentioned hypothesis that HIV-positivity contributed to less exposure to sexual risk factors in our study population is indirectly supported by the magnitude of this adjusted *OR* (corrected for age, employment and sexual risk factors), which was closer to the null value of one than the crude *OR*. The fact that HIV-negative women had an increased chance of being infected with STIs might be explained by the lack of sexual health care programmes targeted at this population. This highlights the need to develop an appropriate education and screening programme for STIs during antenatal care in Gabon for all pregnant women, irrespective of HIV status. In our study population, special counselling by health care providers (e.g. education on condom use and sexual abstinence) exclusively targeted HIV-positive women due to being recommended by the Gabonese National HIV/AIDS Control Programme. Extending such interventions to all pregnant women could positively impact maternal and child health and reduce maternal morbidity and, consequently, neonatal morbidity and mortality [4]. Furthermore, it seems essential to further investigate the impact of behavioural factors on concomitant infections and STIs in HIV-positive and HIV-negative pregnant women in Gabon, particularly by conducting longitudinal studies.

Despite the strong evidence for an association between HIV-positivity and lower crude odds of concomitant STIs (*P* = 0.009), this result should be viewed with caution. Maternal age in the HIV-negative sub-sample was lower than in the HIV-positive sample. Studies have revealed that a younger maternal age may increase the risk of getting STIs and may underline the findings described in this study [12]. However, when adjusting only for age, we cannot say that age has affected the odds of acquiring STIs did not change significantly in our study population, since even after adjustment the statistical association remained still robust (*OR* adjusted only for age = 0.42, *P* = 0.01). However, when adjusting for all other confounders (i.e. mainly sexual risk factors), the negative association between HIV-positivity and concomitant STIs was lost (*OR* = 0.57; *P* = 0.28); as mentioned above, this indirectly supports the role of sexual risk factors in the association between presumable protection of HIV against concomitant STIs.

A strength of this study is that we recruited all pregnant women who tested positive for HIV during their ANC unit visit; this was feasible in our target region, as Gabon is an HIV low prevalence country. Also, for feasibility reasons, only a sub-sample of all HIV-negative pregnant women was recruited. While this may have caused selection bias, our ‘frequency matching’ criteria were successful, indicating a low risk of selection bias. Furthermore, we cannot exclude that very sick HIV-positive pregnant women may have stayed at home and were not present at the ANC unit; such cases would not have been included in our study. Also, it was not possible to acquire all diagnostic samples from all participants resulting in a certain proportion of missing data for each evaluated co-infection; however, the proportion of missing data was similar between HIV-positive and HIV-negative women suggesting again a low risk of selection bias. Furthermore, in this study, we did not comprehensively acquire behavioural confounding factors, so that residual confounding may play a role.

As this is an exploratory study that did not involve a priori sample size calculations, it may be possible that our analysis was prone to alpha error, meaning that the association between HIV-positivity and decreased odds for STIs may constitute a false-positive finding that would not be found in a significantly larger study sample. Further limitations could have been the performance of the different diagnostic methods. PCR has increased sensitivity and specificity compared to rapid diagnostic tests, is used for routine diagnostics like TP and HBV and is known to be more objective than light microscopy. Yet, it is of mention that direct determination of infectious pathogens with PCR often necessitates biological sample testing within specific diagnostic windows; in our study, all women were systematically tested irrespective of individual past medical histories and therefore, some infections may have been missed by PCR testing. Lastly, it is a strength that we applied microscopy reading, the gold standard for diagnosing *Plasmodium* infections; microscopic analytical results were produced exclusively by trained personnel.

## Conclusions

We found a high prevalence of STIs among Gabonese women during pregnancy. HIV-negative women were more often infected with an STI than HIV-positive pregnant women. This may potentially be explained by a comparatively lower favourable sexual risk behaviour of HIV-positive women due to better access to sexual health care education within the framework of the national HIV Control Programme. Better access to sexual health care education for all pregnant women irrespective of HIV status may prove particularly important to contribute towards an improvement of maternal health for all pregnant women.

## Data Availability

The datasets generated during and/or analysed during the current study are not publicly available (because some secondary manuscripts are still being written) but are available from the corresponding author upon reasonable request.

## Abbreviations

AIDS: Acquired immunodeficiency syndrome
ANC: Antenatal care
ART: Antiretroviral treatment
BNITM: Bernhard-Nocht-Institut für Tropenmedizin
CD4: Cluster of differentiation 4
CERMEL: Centre de Recherches Médicales Lambaréné
*CI*: Confidence interval
CLIA: Chemiluminescent immunoassay
CTX: Cotrimoxazole
CT: *Chlamydia trachomatis*
DNA: Deoxyribonucleic acid
EBV: Epstein-Barr-virus
ELISA: Enzyme-linked immunosorbent assay
GBS: Group B *Streptococcus*
HBV: Hepatitis B virus
HCV: Hepatitis C virus
HEV: Hepatitis E virus
HIV: Human immunodeficiency virus
HPV: Human papillomavirus
HTVL: Human T-lymphotropic virus
ICH-GCP: International Conference on harmonization of Technical Requirements for registration of pharmaceuticals for human use—Good clinical Practice
IgG: Immunoglobulin G
IgM: Immunoglobulin M
IQR: Interquartile range
MTCT: Mother-to-child-transmission
NG: *Neisseria gonorrhoea*
*OR*: Odds ratio
PCR: Polymerase chain reaction
*SD*: Standard deviation
SSA: Sub-Saharan Africa
STI: Sexually transmitted infections
TP: *Treponema pallidum*
TV: *Trichomonas vaginalis*
UMCHE: University Medical Centre Hamburg-Eppendorf
UN: United Nations
UNAIDS: United Nations programme on HIV and AIDS
WHO: World Health Organization
